# The Role of GABA in Human Motor Learning

**DOI:** 10.1016/j.cub.2011.01.069

**Published:** 2011-03-22

**Authors:** Charlotte J. Stagg, Velicia Bachtiar, Heidi Johansen-Berg

**Affiliations:** 1 Oxford Centre for Functional Magnetic Resonance Imaging of the Brain (FMRIB), Department of Clinical Neurosciences, University of Oxford, Oxford OX3 9DU, UK

## Abstract

GABA modification plays an important role in motor cortical plasticity [1–4]. We therefore hypothesized that interindividual variation in the responsiveness of the GABA system to modification influences learning capacity in healthy adults. We assessed GABA responsiveness by transcranial direct current stimulation (tDCS), an intervention known to decrease GABA [5, 6]. The magnitude of M1 GABA decrease induced by anodal tDCS correlated positively with both the degree of motor learning and the degree of fMRI signal change within the left M1 during learning. This study therefore suggests that the responsiveness of the GABAergic system to modification may be relevant to short-term motor learning behavior and learning-related brain activity.

## Highlights

► Change in GABA due to transcranial stimulation correlates with motor learning behavior ► GABA change also correlates with localized fMRI responses during short-term learning ► No correlations are found for these measures with GABA levels in visual cortex

## Results

There is considerable variability in motor learning behavior across individuals [7], and the present study aimed to test whether some of this variability could be explained by variation in responsiveness of the GABA system, because GABA modulation plays an important role in learning [1–4]. As a measure of GABA responsiveness, we used magnetic resonance spectroscopy (MRS) to quantify changes in GABA concentration following anodal transcranial direct current stimulation (tDCS), a noninvasive technique that decreases GABA within the motor cortex [5], increases cortical excitability [8], and enhances short-term learning [9]. We predicted that individuals who show less tDCS-mediated GABA modulation would show less behavioral evidence of motor learning and less modulation of fMRI responses during learning.

Subjects participated in three experimental sessions on different days. The first two sessions were MRS sessions, during which GABA-edited spectra were acquired before and after 10 min of tDCS. In the third session, subjects performed an explicit sequence learning task during fMRI, and no tDCS was applied.

### Motor Behavior

Motor learning was assessed via change in reaction times to a visually cued explicit sequence learning task performed with the four fingers of the right hand during fMRI acquisition in session 3.

**Figure 1:**
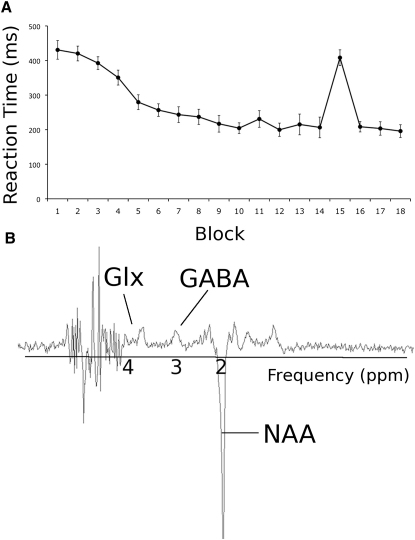
Group Mean Reaction Times in Response to an Explicit Sequence Learning Task and a Typical GABA-Optimized Spectrum (A) Group mean reaction time data showing a decrease in reaction times over blocks as the subjects learned the sequence. Blocks 1 and 15 are blocks containing 30 visual cues in a random order, whereas other blocks contained three repetitions of the same sequence. Points are mean ± standard error of the mean. (B) Typical GABA-optimized spectrum showing characteristic peaks for GABA, Glx, and NAA. See also Figure S1.

All subjects showed a significant reduction in reaction times across successive learning blocks (Figure 1A; repeated-measures analysis of variance, main effect of BLOCK F(15,150) = 19.95; p < 0.001), and further interrogation of the data confirmed that improvements in reaction times occurred primarily via learning of a specific sequence rather than generic skill learning (see Supplemental Data available online). A motor learning score was calculated for each subject as a percentage change from the reaction times in the first sequence block to blocks 10–14 (when performance reached a plateau), and a (nonlearning) motor performance score was calculated as the mean reaction time from the random block (see Supplemental Data).

### Correlation between Behavioral and Neurotransmitter Measures

GABA-optimized MRS was performed before and immediately after 10 min of anodal tDCS during sessions 1 and 2. We performed two scans a week apart, one with a voxel of interest in primary motor cortex (M1) and one with a control voxel in the visual cortex (see Experimental Procedures and Figures S1A and S1B). Resolved peaks were obtained for both GABA, a combined measure of glutamate and glutamine (Glx), and N-acetylaspartate (NAA) (Figure 1B).

**Figure 2:**
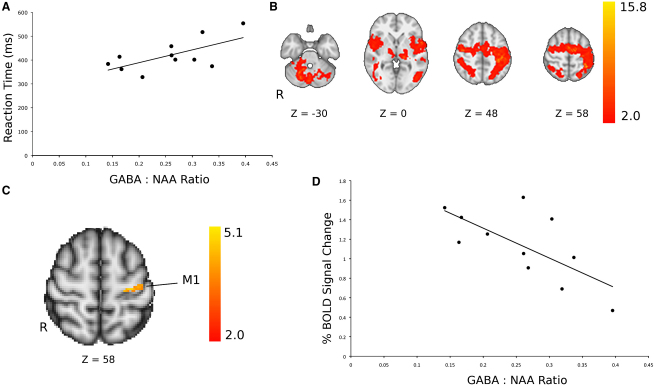
Baseline Measures (A) Significant positive correlation between baseline GABA:NAA ratio within the M1 voxel and reaction times during random (nonlearning) blocks (r = 0.64, p = 0.03). (B) Group mean activation map in response to the motor boxcar regressor demonstrating activity in a bilateral frontoparietal network. Color bar shows Z statistic values. (C) The left primary sensorimotor cortex showed a significant negative correlation between BOLD signal change in response to the motor boxcar and baseline GABA:NAA ratio. (D) Negative correlation between baseline GABA:NAA ratios and mean BOLD signal change in response to the motor boxcar regressor within a left M1 ROI (r = −0.688, p = 0.01, uncorrected). For full details on how the ROI was derived, see Supplemental Experimental Procedures. See also Figure S2.

We first tested for a relationship between baseline neurotransmitter concentrations (prior to tDCS) and metrics of motor performance. There was a significant positive correlation between mean reaction time during random blocks, our measure of motor performance, and the baseline M1 GABA:NAA ratio, such that subjects with a higher GABA:NAA showed slower reaction times (r = 0.64, p = 0.03, uncorrected; Figure 2A). There were no relationships between baseline motor cortex GABA:NAA and behavioral motor learning scores, nor between behavioral measures and baseline motor cortex Glx:NAA or between behavioral measures and any baseline neurotransmitter measures taken from the control voxel in the visual cortex. Because these correlations were not corrected for multiple comparisons, we additionally used a multiple linear regression approach that confirmed the specificity of the relationship (Supplemental Data). Consistent with our previous report [5], anodal tDCS led to a decrease in GABA:NAA of 11.5% ± 4.6% in the M1 voxel (prestimulation GABA:NAA ratio 0.256 ± 0.02; poststimulation GABA:NAA ratio 0.227 ± 0.02; p = 0.04). Baseline GABA:NAA ratio did not predict the induced change in GABA:NAA (r = −0.23, p = 0.48).

**Figure 3:**
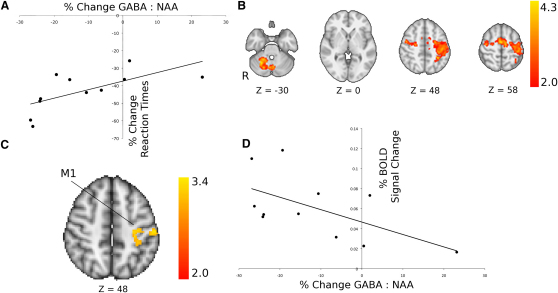
Change Measures (A) Significant positive correlation between change in GABA:NAA ratio due to anodal tDCS and change in reaction times due to learning (r = 0.645, p = 0.03). (B) Group mean activation map in response to the learning regressor demonstrating activity in a more limited bilateral frontoparietal network than that evident in response to the motor boxcar regressor (cf. Figure 2B). Color bar shows Z statistic values. (C) One cluster in left primary motor cortex showed a negative correlation between learning-related change in fMRI activity and change in GABA:NAA ratios due to anodal tDCS. (D) Negative correlation between change in GABA:NAA ratios due to anodal tDCS and the learning-related change in fMRI activity in the left M1 ROI (arbitrary units; r = −0.59, p = 0.05, uncorrected). For full details on how the ROI was derived, see Supplemental Experimental Procedures. See also Figure S2.

We interpret this change in GABA:NAA in response to tDCS as a measure of the responsiveness of the GABA system. Our primary aim was to test whether the responsiveness of the GABA system correlated with behavioral measures of explicit sequence learning. We identified a significant correlation between reaction time change with motor sequence learning and GABA:NAA change in the left M1 due to tDCS (r = 0.645, p = 0.03, uncorrected; Figure 3A). This relationship remained significant after accounting for baseline GABA:NAA, and no relationship was found between reaction time change and baseline GABA:NAA (Supplemental Data). No significant changes with tDCS or correlations with behavior were found for other metabolite measures in M1 or for GABA:NAA measures from the control voxel in the visual cortex, either taken individually or using a multiple linear regression approach (Supplemental Data).

### Correlation between fMRI Measures and Neurotransmitter Measures

Performance of a visually cued explicit sequence learning task during fMRI acquisition on a separate day was associated with activation of a bilateral frontoparietal network (Figure 2B). We performed a voxel-wise test for correlation between baseline M1 GABA:NAA ratio and BOLD signal change between movement blocks versus rest (the motor performance contrast; see Experimental Procedures). After thresholding, one cluster within the left sensorimotor cortex demonstrated a significant negative correlation, such that subjects with a lower baseline GABA:NAA ratio showed greater task-related BOLD responses (Figure 2C; Table 1). These findings were confirmed with region of interest (ROI)-based analyses performed using comparable methods to those used by previous studies (Figure 2D; Figures S2A–S2C). There were no correlations between BOLD signal change for movement performance contrast and baseline M1 Glx:NAA, GABA:NAA, or Glx:NAA in the control visual cortex voxel.

Learning-related changes in task-related fMRI activity were modeled by a contrast-defining reduction in fMRI activity between block 2 and blocks 10–14 (see Experimental Procedures). A voxel-wise test identified a significant negative correlation between learning-related change in fMRI activity and tDCS-mediated change in GABA:NAA ratio within the left M1, such that the greater the decrease in GABA:NAA due to anodal tDCS, the greater the reduction in fMRI activity at this location (Figure 3C). This finding was confirmed using ROI analyses (Figure 3D; Figure S2D).

There were no regions of positive correlation between learning-related change in fMRI activity and M1 Glx:NAA change or change in GABA:NAA or Glx:NAA within the control occipital voxel.

## Discussion

This study aimed to test whether the responsiveness of the GABAergic system to modulation (here achieved through brain stimulation) was associated with variation in short-term motor learning behavior and in learning-related brain activity change. A positive correlation was observed between tDCS-induced GABA decrease in primary motor cortex (M1) and degree of motor learning, such that subjects who demonstrated a greater decrease in M1 GABA following stimulation to M1 also showed faster short-term learning. This finding is in line with the hypothesis that LTP-like plasticity within the neocortex is critically dependent on GABA modulation [10–12]. A negative correlation was also demonstrated between GABA change and learning-related change in fMRI activity within the left M1, such that the greater the decrease in GABA induced by tDCS, the greater the learning-related reduction in fMRI activity. This observation supports the notion that functional plasticity measured with fMRI reflects GABAergic modulation. The functional and anatomical specificity of this relationship is underlined by the finding that, despite testing for a relationship between GABA modulation in M1 and learning-related fMRI change across the whole motor network, the only region showing a significant correlation was localized within the hand area of the primary motor cortex [13].

A number of animal studies have demonstrated that a decrease in tonic GABA is essential for LTP-like plastic changes to be inducible within the motor cortex [10–12]. In humans, a decrease in GABA induced via an ischemic forearm block [14] results in a facilitation of LTP-like plasticity, observed as an increase both in the degree of motor learning [1] and in the response to facilitatory repetitive transcranial magnetic stimulation [15]. In addition, decreases in GABA concentration have been demonstrated using MRS during motor learning [4] and in the acute phase of recovery after stroke [16].

Distinct relationships were found with measures of baseline GABA concentrations. Subjects with higher baseline levels of M1 GABA had slower reaction times and smaller task-related BOLD signal change in left M1. These findings are consistent with previous reports in the occipital cortex, where BOLD signal change in response to a simple visual stimulus correlated with GABA measurements from the primary visual cortex [17]. The results presented here suggest that this relationship is generalizable beyond the visual cortex, and, further, the lack of correlations between GABA concentration in our visual cortical control voxel and any fMRI or behavioral measures suggests that these relationships are specific to task-relevant cortical areas.

### Methodological Considerations

MRS allows accurate quantification of the concentration of neurochemicals within a defined area of cortex, but it does not give direct information as to the synaptic activity within that area and cannot determine where, within the voxel, the changes in neurochemicals occur. However, the close relationship between perisynaptic GABA concentration and vesicular release [18], and the finding that change in MRS-assessed GABA via pharmacological and physiological interventions is associated with a facilitation of LTP-like plasticity [1, 4], suggests that measures of GABA concentration have functional relevance.

This study reports on correlations between GABA and behavioral or functional measures, but it cannot infer causality. Further work should aim to relate GABA decrease during learning with the degree of learning achieved, although this is a technically challenging proposition. In addition, a study designed to test the relationship between tDCS-induced changes in GABA during learning and tDCS-induced changes in behavior during learning would make a stronger case for the causality of the relationship between tDCS-induced GABA change and learning demonstrated here.

It may be that our measure of GABA is a surrogate marker for other changes, such as glutamatergic modification, which in turn may be highly correlated with GABAergic change. Although we find no correlations with our MRS measure of Glx, it should be noted that in our experience MRS measures of Glx appear to have lower sensitivity than GABA measures and in particular are not sensitive to changes in glutamate receptor activity or density, important determinants of neocortical plasticity. In addition, a number of neuromodulators, such as dopamine, serotonin, and acetylcholine, are highly relevant in motor learning, and MRS is insensitive to changes in these chemicals. However, taken together with previous pharmacological studies showing modulation of practice-dependent plasticity with GABA modulation [2, 3] and the animal literature demonstrating the relevance of GABA to learning [19, 20], we believe that the evidence presented here suggests that GABA is important in motor learning in healthy adults.

Because we wished to investigate the relationship between the responsiveness of the GABA system to modulation by tDCS and motor learning, we did not include a sham stimulation condition, but rather included a condition using a control voxel. A previous study using identical stimulation and MR parameters showed that there is no modulation of GABA with sham stimulation [5].

### Conclusions

The finding that the responsiveness of the GABA system to modulation has a strong relationship with motor learning is suggestive of a possible relevance of GABA in an LTP-like synaptic plasticity in human motor learning. In addition, it offers a strong rationale for the use of interventions, such as anodal tDCS, that decrease GABA in a localized region in conjunction with rehabilitation in the context of stroke, in line with recent findings in an animal model [21].

## Experimental Procedures

Twelve volunteers (six male; mean age 23 yr, range 21–31 yr) gave informed consent to participate, in accordance with local ethics committee approval. All subjects were right-handed, as assessed by the Edinburgh Handedness Inventory [22], and had no history of neurological or psychiatric disorder. Subjects participated in three testing sessions on different days. GABA changes with learning have been demonstrated to occur, but only after 50 min of performance of a complex visuomotor task [4]. Because of time constraints, it was therefore not possible to acquire spectra before and after a learning task, so we used tDCS to modulate GABA activity.

### Sessions 1 and 2: tDCS and GABA MRS

We performed two separate MRS studies on a 3T Siemens/Varian MRI System. Subjects lay at rest in the scanner, and MRS data was acquired before and after a 10 min period of tDCS. Detailed information on MRS acquisition is provided in the Supplemental Experimental Procedures. In brief, a voxel of interest was placed over the hand motor cortex in session 1 and the visual cortex in session 2 (Figures S2A and S2B). A standard PRESS sequence was acquired to assess the creatine and NAA line widths, and a MEGA-PRESS sequence was then acquired to allow simultaneous spectral GABA editing, three-dimensional voxel localization, and water suppression [23]. A baseline 256-acquisition GABA-optimized spectrum lasting approximately 15 min was acquired prior to stimulation, and a 384-acquisition GABA-optimized spectrum lasting approximately 20 min was acquired immediately after stimulation. Including setup time, sessions lasted approximately 75 min in total. A representative spectrum is shown in Figure 1B.

For tDCS stimulation, a DC Stimulator (Eldith GmbH; Germany) delivered a 1 mA current to the brain via two electrodes measuring 5 × 7 cm (Easycap, GmbH; Germany). For both sessions, one electrode was placed over the left M1 and centered 5 cm lateral and 2 cm anterior to Cz over the left hemisphere, and the other was placed over the contralateral supraorbital ridge, an electrode configuration that has previously been demonstrated to be effective in modulating motor cortical excitability [8]. The current had a ramp-up time of 10 s, was held at 1 mA for 10 min, and was then ramped down over 10 s.

MRS analysis was performed using the jMRUI software package version 2.2 (http://www.mrui.uab.es/mrui) (Supplemental Experimental Procedures). To allow for any changes in tissue water after stimulation, we give all neurotransmitter concentrations as a ratio to NAA, because no simultaneous creatine peak is acquired. The percentage change in neurotransmitter (NT) concentrations after tDCS was calculated as follows: ([(NT_POST_ – NT_PRE_) / NT_PRE_] × 100%).

### Session 3: Functional MRI and Explicit Learning Task

Details of the fMRI paradigm, image acquisition, and behavioral and image analysis are given in the Supplemental Experimental Procedures. In brief, subjects performed a visually cued reaction time task during echo planar imaging acquisition. The task included sequence blocks that consisted of three repeats of a ten-digit sequence that subjects learned explicitly. The first and fifteenth blocks consisted of 30 visual cues presented in a random order. tDCS has previously been demonstrated to modulate learning in this task (unpublished data). Correlations between neurotransmitter levels and behavioral measures are uncorrected for multiple comparisons.

Image analysis was performed using tools from the FMRIB Software Library (http://www.fmrib.ox.ac.uk/fsl) [24]. After standard preprocessing, statistical analysis was carried out using FMRIB's improved linear model with local autocorrelation correction [25]. Our first-level model for each subject consisted of 18 regressors, with each regressor representing a single task block. Two lower-level contrasts were created from this model: (1) a contrast defining mean activation across the task (i.e., with a weight of 1 assigned to each task block regressor) to model motor performance, and (2) a contrast defining decreased activity between block 2 (the first learning block) and blocks 10−14 (the plateau of performance; i.e., with a weight of 1 assigned to the regressor for block 2 and with weights of −0.2 assigned to each of blocks 10–14) to model learning-related activity.

A second-level mixed-effects analysis was performed to test for voxel-wise correlations between specific lower-level contrasts and GABA measures across individuals. Correlations between baseline GABA:NAA ratio and BOLD signal change in response to the motor performance contrast were masked post hoc by the group mean activation in response to the motor performance contrast. Correlations between GABA:NAA change after tDCS and BOLD signal change in response to the learning regressor were masked post hoc by the group mean activation in response to the learning contrast. For group-level analyses, Z (Gaussianized T/F) statistic images were thresholded using clusters determined by Z > 2.0 and a (corrected) cluster significance threshold of p = 0.01.

## Figures and Tables

**Table 1:** fMRI Activations

Region	Max Z Statistic	Volume (mm^3^)	Coordinates		
			x	y	z
**Negative correlations between boxcar-related BOLD signal change and GABA**					

Left primary sensorimotor cortex	5.11	576	−40	−30	46

**Positive correlation between learning-related BOLD signal change and tDCS-induced GABA change**					

Left primary motor cortex	3.45	1015	−42	−24	64

Negative correlations between the boxcar-related BOLD signal change and GABA refer to areas where subjects who had a higher resting GABA showed smaller BOLD signal change. Negative correlations between the learning-related BOLD signal change and tDCS-induced GABA change refer to areas in which subjects who showed a greater decrease in GABA in response to tDCS also had a greater learning-related BOLD signal change.

## Supplemental Information

Document S1. Two Figures, Supplemental Data, and Supplemental Experimental Procedures
