# Supplementary material for: The Role of GABA in Human Motor Learning
Source: Curr Biol. 2011 Mar 22;21(6):480–4. doi: 10.1016/j.cub.2011.01.069 (PMC3063350; doi:10.1016/j.cub.2011.01.069)
Supplement: Document S1. Two Figures, Supplemental Data, and Supplemental Experimental Procedures [file mmc1.pdf]

Current Biology, Volume *21*

Supplemental Information

The Role of GABA

in Human Motor Learning

Charlotte J. Stagg, Velicia Bachtiar, and Heidi Johansen-Berg

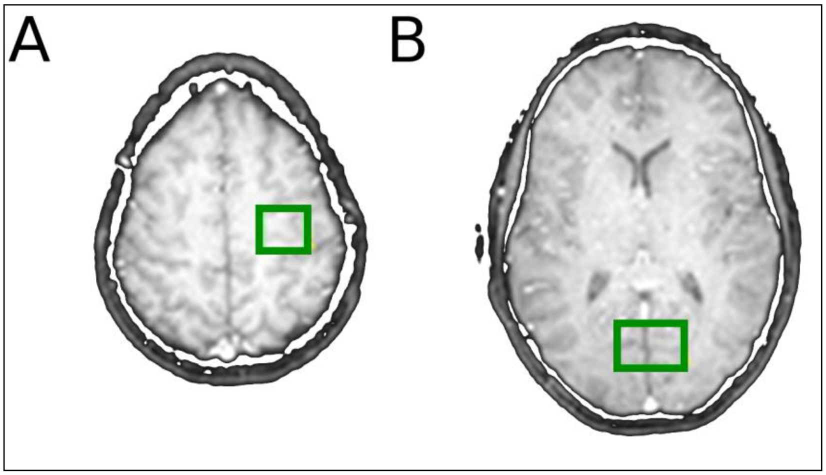

Figure S1. MRS Voxel Locations, Related to Figure 1
(A) Typical placement of the 2x2x2cm voxel within the left sensorimotor cortex.
(B) Typical placement of the 2x3x2cm voxel.

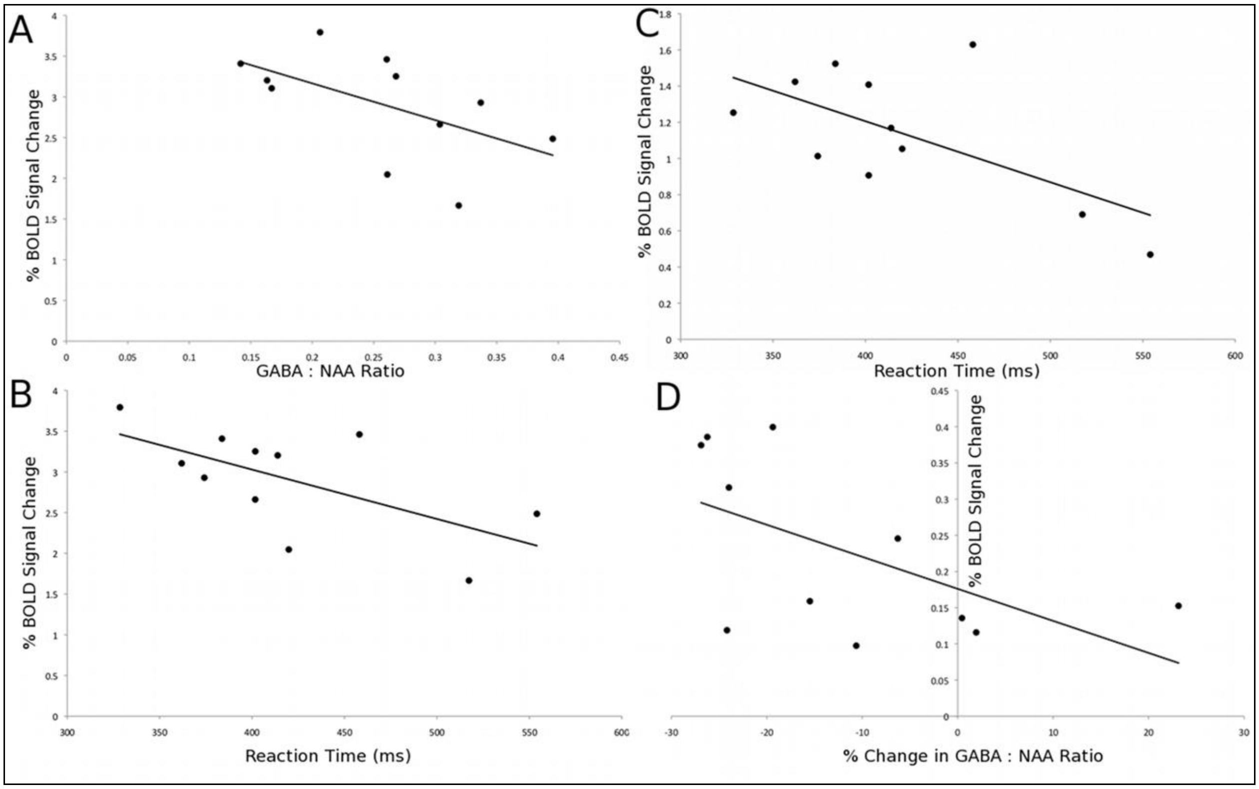

Figure S2. Additional ROI Analyses, Related to Figure 2

(A) ROI analysis demonstrating a negative correlation between baseline GABA:NAA ratios and mean BOLD signal change in response to the motor boxcar regressor within the maximally activating voxel within the left M1 ($r = -0.585$, $p = 0.05$).

(B & C) ROI analysis demonstrating a negative correlation between motor performance related BOLD signal change in M1 and baseline reaction time (B: maximally activating voxel $r = -0.633$, $p = 0.03$; C: mask $r = -0.629$, $p = 0.03$). For full details on how ROIs were derived see supplemental experimental procedures.

(D) ROI analysis demonstrating a negative correlation between the learning-related change in fMRI activity in the maximally activating voxel within M1 and change in GABA:NAA ratio ($r = -0.571$, $p = 0.06$).

Supplemental Data

Motor Behaviour

All subjects showed a significant reduction in reaction times across successive learning blocks (Figure 1, repeated measures ANOVA, main effect of BLOCK $F(15,150)=19.95$; $p<0.001$). By contrast, there was no significant difference in mean reaction time between the two random blocks [Block 1: (mean ± S.E.) 430±27ms; Block 15: 408±23ms; paired t-test, $t=0.75$; $p=0.4$], whereas there was a significant difference between Block 14 (the final learning block) and Block 15 (the second random block); (paired t-test $p<0.001$) suggesting that improvements in reaction times occurred via learning of a specific sequence and not generic skill learning.

There was no significant difference between the reaction times from blocks 10-14, which were on the plateau of the learning curve ($F(4,40)=0.538$, $p=0.7$). Therefore, a mean reaction time from blocks 10 to 14 was calculated for each subject and this was used to calculate a percentage change from the reaction times in the first sequence block to give a measure of motor learning (Block 2) (i.e. $[(RT_{(10\text{-}14)} - RT_{(2)}) / RT_{(2)}] * 100\%$). Reaction times decreased from 420±21.4ms in block 2 to 211±19.9ms in blocks 10-14, a decrease of 48.2±5.6 % (t-test $p<0.001$). There was no significant correlation between the initial reaction time (Block 1 mean) and the change in reaction time with learning ($p > 0.1$).

Baseline Measures

Correlation between Motor Performance and Baseline Neurotransmitter Concentrations

The mean baseline GABA:NAA ratio within left M1 was 0.25±0.02 and the mean baseline Glx:NAA ratio 0.36±0.05. The baseline levels of the two neurotransmitters were significantly positively correlated with each other ($r=0.68$, $p=0.02$). There was a significant positive correlation between mean reaction time during random blocks, our measure of motor performance, and the baseline M1 GABA:NAA ratio, such that subjects with a higher GABA:NAA ratio showed slower reaction times ($r=0.64$, $p=0.03$) (Figure 2A). There was no relationship between motor performance and Glx:NAA ($r=0.258$, $p=0.4$). Since GABA:NAA and Glx:NAA covary, multiple linear regression was performed to determine relative contributions of these two neurotransmitters to reaction times. The correlation between GABA and RT remained significant ($\beta=0.81$, $p=0.05$) and there was no correlation between Glx and RT ($\beta= 0.294$, $p=0.44$).

To assess the anatomical specificity of this relationship, we assessed GABA:NAA and Glx:NAA ratios from the control voxel within the V1 cortex. The mean baseline GABA:NAA ratio in the V1 cortex was 0.259±0.01 and the mean Glx:NAA ratio was 0.49±0.02. There was no correlation between the neurotransmitter:NAA ratios in the V1 voxel and those in the M1 voxel (GABA $r=-0.09$, $p=0.82$; Glx $r=-0.21$, $p=0.57$) and no correlation between neurotransmitter:NAA ratios in the V1 voxel and mean reaction time during the random blocks (GABA $r=- 0.10$, $p=0.78$; Glx $r=0.15$, $p=0.66$). Confirming the anatomical specificity of the relationship between reaction times and baseline GABA to M1, correlations with the M1 voxel were found to be significantly stronger than those for the V1 voxel (M1 GABA:RT $r=0.64$, V1

GABA:RT r=-0.10, Fisher's r-to-Z transform to test for difference (http://faculty.vassar.edu/lowry/VassarStats.html), Z=1.72, p=0.04).

Change Measures

Correlation between Learning and GABA Modulation by tDCS

As reported in the main text, we found a correlation between the change in GABA:NAA evoked by tDCS and change in RT with motor learning. No GABA or RT values were identified as significant outliers (Grubbs' Test at $\alpha = 0.05$; critical Z=2.35; GABA: highest calculated Z=1.72, RT: highest calculated Z=2.01; no outliers detected; www.graphpad.com/quickcalcs/grubbs). There was no significant correlation between baseline GABA and reaction time change ($r = -0.2$, $p > 0.9$) and the correlation between the change in GABA and reaction time change remained significant when multiple linear regression was performed to account for baseline GABA levels ($r=0.67$, $p = 0.03$).

There was no significant change in Glx following tDCS [pre-stimulation Glx:NAA ratio 0.362±0.06; post-stimulation 0.389±0.06; p=0.37], nor was there a significant correlation between change in GABA:NAA and change in Glx:NAA (r=-0.16, p=0.63). There was also no change in the reference peak NAA:Creatine after stimulation [pre-stimulation ratio 2.29±0.08; post stimulation 2.31±0.11; p=0.87].

To test the anatomical specificity of tDCS-induced changes we investigated the change in GABA:NAA ratios in the control V1 voxel after anodal tDCS applied to the M1. There was no significant change in GABA:NAA within the V1 voxel (change -2.7±3.56% [pre-stimulation GABA:NAA ratio 0.259±0.01; post-stimulation 0.250±0.01; p=0.4]) and there was no correlation between change in GABA within the V1 voxel and reaction time change with motor sequence learning (r=-0.17, p=0.63). The anatomical specificity of the relationship between motor sequence learning and GABA was again confirmed by demonstrating that correlations with M1 were significantly stronger than those for V1 (M1 ΔGABA : ΔRT r = 0.645, V1 ΔGABA : ΔRT r = -0.17, Fisher's r-to-Z transform to test for difference (http://faculty.vassar.edu/lowry/VassarStats.html), Z = 1.88, p = 0.03).

Multiple Linear Regression

In order to confirm the specificity of the relationships between GABA and measures of RT demonstrated in this paper we performed a multiple linear regression analysis, modelling baseline reaction time and change in reaction time in separate models. In each model we included baseline GABA:NAA; tDCS-induced change in GABA:NAA; baseline Glx:NAA; and tDCS-induced change in Glx:NAA as predictive variables. MRS measures from M1 and V1 measures had to be included in separate models due to insufficient degrees-of-freedom in the data. This approach confirmed the relationships described in the text i.e. that baseline reaction times were correlated with baseline GABA:NAA within M1; change in reaction times was correlated with tDCS-induced change in GABA:NAA; and no other relationships between MRS measures and behavioural measures were significant.

Models based on MRS Measures from M1

Predicting Baseline Reaction Times: M1 Baseline GABA:NAA β=1.05, p=0.01; Change in M1 GABA:NAA β=-0.03, p>0.8; Baseline M1 Glx:NAA β=-0.713, p>0.1, Change in M1 Glx:NAA β=-0.636, p>0.1.

Predicting Change in Reaction Times with learning: M1 Baseline GABA:NAA β=0.463, p=0.1; Change in M1 GABA:NAA β=4.70, p=0.003; Baseline M1 Glx:NAA β=-0.519, p>0.08, Change in M1 Glx:NAA β=-0.268, p>0.2.

Models based on MRS measures from V1

Predicting Baseline Reaction Times: V1 Baseline GABA:NAA β=0.309, p>0.3; Change in V1 GABA:NAA β=0.210, >0.5; Baseline V1 Glx:NAA β=0.749, p>0.1, Change in V1 Glx:NAA β=0.816, p>0.06

Predicting Change in Reaction Times with learning: V1 Baseline GABA:NAA β=0.335, p>0.4; Change in V1 GABA:NAA β=-0.269, >0.6; Baseline V1 Glx:NAA β=-0.006, p>0.9, Change in V1 Glx:NAA β=0.002, p>0.9

Supplemental Experimental Procedures

tDCS Stimulation

For use in the scanner, electrodes were fitted with 5kΩ resistors sited next to the electrode pad to minimise the possibility of eddy currents induced in the leads during MRS leading to heating under the electrodes (for full details see [5]). tDCS leads were positioned parallel to the subject, not touching the skin, and care was taken to ensure that the two leads did not come in to contact at any point. As reported previously, no distortion in images was seen using this experimental setup [5].

High Chloride EEG electrode paste (Easycap,GmbH; Germany) was used as a conducting medium between the scalp and the electrodes. During MRS acquisition the electrodes were unplugged from the stimulator. For stimulation, extension leads connected the stimulator, located outside the magnetic field, to the subject within the bore of the magnet. After stimulation was complete, leads were disconnected from the stimulator and acquisition recommenced. No MRS data were acquired during tDCS.

MRS Acquisition

A 3T Siemens/Varian MRI system was used. In both MRS sessions sagittal and axial T1-weighted scout images were acquired and used to place the voxel of interest. In session 1, a 2x2x2cm voxel of interest was placed manually over the left precentral knob, to target the hand motor representation [13] as in previous studies [4, 5] (Supplementary Fig 2A). We performed a separate MRS session with a 3x2x2cm control voxel of interest placed manually in the occipital cortex, centred on an axial slice drawn though the AC-PC line (Supplementary Fig 2B).

To assess the creatine and NAA linewidths, a standard PRESS sequence [TR = 3s, TE = 68ms] was used to acquire an unedited spectrum with 32 averages. Water was suppressed at 4.7ppm using a method similar to WET [S1].

The MEGA-PRESS sequence [TR = 3s, TE = 68ms] was then used to allow simultaneous spectral GABA editing, three-dimensional voxel localisation and water suppression [23]. A selective double-banded 180° pulse was created from 20-ms Gaussian pulses. The frequency of the first band of this pulse was set to 4.7 ppm to suppress water. The second band was alternated between 1.9 ppm, the resonance frequency of C3 protons (strongly coupled to the observed C4 GABA protons and 3.0 ppm; condition A), and 7.5 ppm, which is symmetrically disposed about the water resonance to equalize off resonance effects (condition B). The resonance at 1.9 ppm was inverted 180° during condition A but not during condition B. In condition A, the GABA C4 (triplet) resonance (at 3.0 ppm) therefore was fully refocused, whereas in condition B, this peak was not refocused, but phase modulated so that the outer triplet signals were inverted at echo time TE = 68 ms. The difference spectra from conditions A and B (at TE = 68 ms) revealed the edited GABA spectrum without the larger overlapping creatine resonance. A representative spectrum is shown in Figure 1B.

MRS Analysis

The free induction decay signal (FID) was first corrected for any non-zero DC offset and the signal was smoothed using a 2Hz Lorentzian filter. The residual water signal was then filtered out using Singular Value Decomposition techniques. The spectrum was then phased with respect to both the 0th and 1st order phase.

The non-edited PRESS acquisition was analysed using AMARES, a non-linear least square fitting algorithm operating in the time domain [S2]. Peak fitting for NAA and creatine was performed using Gaussian curves to obtain linewidths for these resonances.

The GABA-optimised spectra were then analysed using AMARES, as above. Peak fitting for the GABA and glutamate/glutamine resonances was performed using Gaussian curves with the linewidth constrained to that of the creatine resonance in the non-edited spectrum. The GABA and the glutamate/glutamine resonances were both fitted with 2 Gaussian peaks. The linewidth of the inverted NAA resonance was constrained to the linewidth of NAA in the unedited spectrum and a single Gaussian curve was fitted to this peak. The amplitudes of both GABA peaks were summed to give a total value for GABA, likewise summing was performed for the Glx peak.

To correct for the expected contribution from mobile brain macromolecules (MM, which include cytosolic proteins) the GABA-nulled spectrum was analysed as above, and area under the peak resonating at 3ppm was calculated. This was then subtracted from the GABA resonance, to give a value representing the contribution from GABA alone.

Analysis was conducted by two independent observers who were blind to whether an individual spectrum was acquired pre- or post-stimulation. The inter-rater reliability co-efficient was α = 0.994.

A T1-weighted structural scan was also acquired during each MRS session (TR = 3s; TE = 5ms, TI = 1ms; FOV 512 x 256, matrix 256 x 128). FMRIB's Automated Segmentation Tool (FAST) [S3] part of the FMRIB software library (www.fmrib.ox.ac.uk/fsl) [24], was used to calculate the relative quantities of grey matter and white matter within the voxel. The amplitude of the GABA and Glx peaks were corrected for the proportion of Grey Matter volume within the voxel (multiplied by [GM]/([GM]+[WM]+[CSF]) and NAA and creatine were corrected for the proportion of total brain tissue volume within the voxel (multiplied by ([GM] + [WM])/([GM]+[WM]+[CSF]).

fMRI Analysis

The following pre-statistics processing steps were applied: motion correction using MCFLIRT [S4]; non-brain tissue removal using BET [S5]; spatial smoothing with a Gaussian kernel of 5mm full-width at half-maximum; mean-based intensity normalization of all volumes by the same factor; nonlinear high pass temporal filtering (Gaussian-weighted least squares straight line fitting with sigma = 50.0 seconds). Fieldmap-based EPI unwarping was performed using

PRELUDE+FUGUE [S6]. Registration to T1-weighted high resolution individual subject anatomical images and to a standard MNI152 template image was carried out using FLIRT [S4].

Region of Interest Analysis

In order to compare our results with a previous report of correlations between GABA levels and FMRI responses within the visual cortex [17], we additionally performed a region of interest (ROI) analysis using similar methods to those employed in the previous study. An ROI for M1 was drawn on the standard MNI152 template image. The mask included the cortex in the anterior bank of the central sulcus plus the posterior half of the precentral gyrus, extending from the level of the dorsal surface of the lateral ventricles to the most dorsal point of the brain, and from the lateral surface of the brain to the interhemispheric fissure. For each subject separately, the peak voxel for activation in response to the motor sequence boxcar regressor within this masked area was identified and the percent BOLD signal change at this voxel was calculated, as reported previously [17]. In addition, as the maximum voxel may be dominated by signal from a blood vessel, rather than from the cortical tissue we also created a 3x3x3 voxel cubic mask centered at those coordinates and the mean BOLD signal change within this mask was then extracted. These peak activations were calculated for both the boxcar regressor and the learning regressor and tested for correlation with baseline neurotransmitter levels and neurotransmitter change respectively.

Separate analyses were performed using the GABA baseline and change values from the control voxel in the V1 cortex in an identical manner.

Supplemental References

S1. Ogg, R.J., Kingsley, R.B., and Taylor, J.S. (1994). WET, a T1- and B1-Insensitive Water-Suppression Method for in Vivo Localized 1H NMR Spectroscopy. J. Magn. Res. B *104* 1-10.

S2. Vanhamme, L., Van den Boogaart, A., and Van Huffel, S. (1997). Improved method for accurate and efficient quantification of MRS data with use of prior-knowledge. J. Magn. Res. *129*, 35-43.

S3. Zhang, Y., Brady, M., and Smith, S. (2001). Segmentation of brain MR images through a hidden Markov random field model and the expectation maximization algorithm. IEEE Trans. Med. Im. *20*, 45-57.

S4. Jenkinson, M., Bannister, P., Brady, J., and Smith, S. (2002). Improved optimisation for the robust and accurate linear registration and motion correction of brain images. Neuroimage *17*, 825-841.

S5. Smith, S. (2002). Fast, Robust Automated Briain Extraction. Hum. Brain Mapp. *17*, 143-155.

S6. Jenkinson, M. (2003). A fast, automated, n-dimensional phase unwrapping algorithm. Magn. Reson. Med. *49*, 193-197.
